# Supplementary material for: Increased perception of the experience dimension of the animal mind reduces instrumental violence against animals
Source: PLoS One. 2023 Nov 30;18(11):e0295085. doi: 10.1371/journal.pone.0295085 (PMC10688919; doi:10.1371/journal.pone.0295085)
Supplement: S1 Appendix — Experimental manipulation. (DOCX) [file pone.0295085.s001.docx]

**Appendix – Pilot Study. Experimental manipulation**

In the pilot study we tested the effectiveness of manipulation.

**Hypothesis**

HP1 In the experimental groups (Experience 1 and Experience 2), the perception of animal experience capacities will be higher than in the control group.

Between September, 2021 and November, 2021, 229 Polish participants were recruited from Facebook. Each participant was randomly assigned to one of three groups: control, Experience 1, Experience 2, and was informed that this study explores the importance of emotions in agricultural management. Next, participants provided their informed consent (before access to the study content) and their sociodemographic data, performed a math task and a manipulation task, completed AMQ-PL, and after that, completed MFQ-PL. Finally, the real goal of the study was revealed.

Before the analyses, the quality of the collected data was verified. We excluded those respondents who answered incorrectly on control items and provided identical answers to all items on at least one of the questionnaires used (*n* = 5), and incorrectly answered on control item (*n* = 15). Since some participants took a long time to complete the survey, we excluded them ((*n* = 41, maximum time was 48 h). The final sample of 165 participants was aged between 18 and 70 years old (M = 31.39, SD = 13.14), 86.7% were women, 83.6% lived in a city, 16.4% lived in in the country.

**Experimental manipulation**

All participants read texts which were assigned to their group (condition). In the control group, the text was about colors. In Experience 1, it was about cows’ and pigs’ capacities to feel pain, fear, hunger. The text assigned to Experience 2 was about cows’ and pigs’ capacities to feel anger, joy, pleasure, and about the animals' personality. Next, the participants were asked to write a short post for Facebook or Twitter about the text they read ((Table S1.). Finally people to completed the Animal Mind Questionnaire (AMQ-PL, Potocka & Bielecki, 2023, based on the Mind Survey, Gray et al., 2007).

**Measures**

**Manipulation check**

We used the AMQ-PL to check manipulation by subscale of experience ( = 0.86), and to control for anthropomorphization, we used the subscale of agency ( = 0.90). The experience subscale measured perception of the experience dimension, with items on hunger, fear, pain, pleasure, rage, desire, personality, consciousness, pride, embarrassment, and joy. The agency subscale (2) measured perception of the agency dimension, with items on morality, memory, emotion recognition, planning, self-control, communication, and thought. Participants were instructed: “Using a scale of 0 (not at all) to 6 (very much), please tell us how capable do you think horses, cows, goats, sheep are of: ”and provided with descriptions of 18 mental capacities from the original scale, e.g., “understanding how others are feeling”, “feeling afraid or fearful”, “telling right from wrong and trying to do the right thing” (see Table S1).

**Controlled variable**

We controlled moral foundations of care and authority. They were measured by MFQ-PL (Jarmakowski-Kostrzanowski & Jarmakowska-Kostrzanowska, 2016), (care = 0.62, authority = 0.72). MFQ-PL includes six items per foundation and is measured on a six-point scale.

Masked task. Participants solved four simple math tasks.

**Results**

First, we calculated means of variables (Table S2). Next, to confirm the effect of manipulation, we calculated contrast tests in OLS model for perception of experience, anthropomorphization and moral foundations of care and authority. We compared the experimental groups with the control group (contrast 1) and the experimental groups with each other (contrast 2) (Table S3).

Results confirmed higher perception of experience in the experimental groups (Experience 1 + Experience 2) versus control, however, this is accompanied by higher anthropomorphization (contrast 1). A contrast test between the experimental groups (contrast 2) revealed no differences between Experience 1 and Experience 2. In both contrasts, there were no differences in both moral foundations.

**Table S1. Manipulation. Instruction for participants and text in 3 experimental conditions.**

| Prosimy Pana/Panią przeczytanie tekstu, a następnie wykonanie krótkiego zadania związanego z przeczytanym tekstem. | | |
| --- | --- | --- |
| control (89 words) | Experience 1 (89 words) | Experience 2 (89 words) |
| **Badania naukowe pokazują, że ludzie preferują pewne kolory w zależności od wieku, płci i nastroju. Dzieci lubią kolory wyraziste i mocne - na przykład intensywny czerwony, żółty lub niebieski. Dorośli natomiast, podczas zakupów ubrań lub mebli, unikają intensywnych barw wybierając, kolory stonowane, takie jak beż, różne odcienie szarości lub zielonego. Podczas gdy kobiety chętnie kupują zarówno produkty różowe, błękitne, pomarańczowe jak i czarne, mężczyźni znacznie częściej wybierają towary czarne, brązowe, szare lub granatowe. Kolory mogą wpływać na poprawę lub pogorszenie nastroju, bardziej optymistyczne myśli lub obniżenie lęku i niepokoju.** | **Badania naukowe pokazują, że świnie i krowy odczuwają głód, ból i strach. Boją się gdy w ich otoczeniu pojawia się nieznany człowiek i próbuje się do nich zbliżyć. Młode prosiaki podczas przycinania ogonów głośno kwiczą, a po zabiegu uciekają i starają się ukryć. Krowy boją się elektrycznego pastucha na pastwiskach ponieważ ból jaki czuja w przypadku porażenia prądem jest dla nich bardzo dotkliwy. Zarówno krowy jak i świnie w przypadku braku pożywienia silnie odczuwają głód. Podczas długiego transportu potrafią zasłabnąć z powodu stresu związanego z brakiem paszy lub wody.** | **Badania naukowe pokazują, że świnie i krowy mogą przeżywać złość lub depresję, być zmartwione lub przybite - świat wydaje się wtedy mniej wesoły i kolorowy. Krowy są dużymi śpiochami – uwielbiają spać, śpią nawet piętnaście godzin dziennie – to duża przyjemność dla nich.** Świnie natomiast bardzo lubią się bawić. Mogą to być zabawy piłką lub w berka z innymi świniami – sprawia im to dużą radość. **Krowy s**ą czułymi matkami, gdy opiekują się cielętami na pastwiskach. Każda krowa czy świnia jest trochę inna, ma cechy które pozwalają odróżniać każdego osobnika w stadzie. |
| Prosimy Pana/Panią o napisanie krótkiej wiadomość do 15 słów, by zaprezentować przeczytane treści na Twitterze lub Facebooku …................................................................................................................................................ | | |

**Table. S2. Descriptive statistics. Means of the examined variables (Pilot Study).** *N* = 165.

| Zmienne | Total | | control  *N* = 59 | | Experience 1  *N* = 52 | | Experience 2  *N* = 63 | |
| --- | --- | --- | --- | --- | --- | --- | --- | --- |
|  | *M* | *SD* | *M* | *SD* | *M* | *SD* | *M* | *SD* |
| Perception of experience  (moral patient) | 4.51 | 0.95 | 4.28 | 1.07 | 4.60 | 0.86 | 4.66 | 0.84 |
| Perception of agency  (anthropomorphization) | 2.66 | 1.40 | 2.36 | 1.54 | 2.93 | 1.25 | 2.73 | 1.32 |
| Care | 5.26 | 0.63 | 5.37 | 0.64 | 5.22 | .64 | 5.20 | 0.60 |
| Authority | 2.67 | 0.93 | 2.74 | 0.82 | 2.57 | .97 | 2.69 | 0.99 |

**Table. S3. Differences in the status of moral recipient, anthropomorphization and moral foundations between groups. Results of contrast tests. (Pilot Study).** *N* = 165. Bold font indicates statistically significant results. Contrast 1 = both experimental groups together (Experience 1 and Experience 2) vs, the control group, contrast 2 = Experience 2 vs Experience 1.

| Zmienne | Contrast 1 | | | Contrast 2 | | |
| --- | --- | --- | --- | --- | --- | --- |
|  | *Difference* | *SE* | *p* | *Difference* | *SE* | *p* |
| Perception of experience  (moral patient) | **0.35** | 0.15 | 0.025 | 0.06 | 0.18 | 0.739 |
| Perception of agency  (anthropomorphization) | **0.47** | 0.23 | 0.041 | −0.20 | 0.27 | 0.468 |
| Care | −0.16 | 0.10 | 0.111 | −0.02 | 0.12 | 0.855 |
| Authority | −0.11 | 0.15 | 0.472 | 0.12 | 0.18 | 0.523 |
