# Supplementary material for: Increased perception of the experience dimension of the animal mind reduces instrumental violence against animals
Source: PLoS One. 2023 Nov 30;18(11):e0295085. doi: 10.1371/journal.pone.0295085 (PMC10688919; doi:10.1371/journal.pone.0295085)
Supplement: Attachment — Submitted filename: PONE-D-23-19973.docx [file pone.0295085.s002.docx]

Dear Editor,

I would like to offer my feedback on the paper titled "Increased perception of the experience dimension of animal mind reduces instrumental violence against animals." Firstly, I'd like to extend my congratulations to the authors for their valuable work and engaging topic. This research paper explores the relationship between the perception of animal minds and instrumental violence against animals. It investigates whether increasing the perception of animals as moral patients with experience capacities reduces violence against them, distinct from the effect of anthropomorphization (Perception of Agency).

I would also like to provide some specific comments on the paper:

1. Abstract: The abstract should offer a concise summary of your study, including the research question, methodology, key findings, and implications. Ensure that it highlights the significance of your research. Make sure your abstract provides a clear overview of the study.
2. Introduction: Clearly state the research problem and research questions in the introduction. Provide a brief literature review to establish the context and importance of your study. Simplify the introduction for better readability.
3. Methods: Explain your experimental design in detail, including participant recruitment, the procedure they followed, and the manipulation task. Provide a clear rationale for your choice of measures and questionnaires.
4. Results: Present your results in a clear and organized manner. Avoid overwhelming the reader with excessive statistical details. Focus on the most critical results that address your hypotheses.
5. Discussion: Discuss the effect sizes and practical significance of your findings. Explore the implications of your findings for the field and for addressing the issue of instrumental violence against animals. Address potential limitations of your study, such as sample size, generalizability, or other methodological concerns. Suggest areas for future research or extensions of your work.
6. Conclusions: Conclude your paper by summarizing the main findings and their significance. Discuss the practical implications of your research and how it contributes to the broader understanding of the topic. Please provide a description of the scientific novelty presented in the study, including a section on the study's limitations and research perspectives in the conclusion.
7. Lastly, it's advisable to include the most recent references, primarily those published between 2017 and 2023, except in the method section where older references might be more relevant.
