# Supplementary material for: Increased perception of the experience dimension of the animal mind reduces instrumental violence against animals
Source: PLoS One. 2023 Nov 30;18(11):e0295085. doi: 10.1371/journal.pone.0295085 (PMC10688919; doi:10.1371/journal.pone.0295085)
Supplement: Attachment — Submitted filename: PONE-D-23-19973_R1.docx [file pone.0295085.s004.docx]

Dear Editor,

I would like to provide my feedback on the paper titled "Increased perception of the experience dimension of the animal mind reduces instrumental violence against animals".

This marks my second set of comments on this engaging scientific paper. The authors have made substantial improvements to the manuscript, addressing the suggestions effectively. I believe the paper now meets the necessary criteria for publication and has significant merit in contributing to the field.

I want to extend my heartfelt appreciation to the authors for their dedicated efforts in enhancing the quality of this paper. Their hard work and commitment to improving the manuscript are greatly acknowledged and valued.
